# Implementation of a UK supermarket intervention to increase purchasing of fresh fruit and vegetables: process evaluation of the WRAPPED natural experiment

**DOI:** 10.1186/s12966-024-01679-3

**Published:** 2024-11-11

**Authors:** Janis Baird, Preeti Dhuria, Hannah Payne, Sarah Crozier, Wendy Lawrence, Christina Vogel

**Affiliations:** 1 Medical Research Council Lifecourse Epidemiology Centre, University of Southampton, Southampton General Hospital, Tremona Road, Southampton, UK; 2 NIHR Southampton Biomedical Research Centre, University of Southampton and University Hospital Southampton NHS Foundation Trust, Tremona Road, Southampton, UK; 3 NIHR Applied Research Collaboration Wessex, Southampton Science Park, Innovation Centre, 2 Venture Road, Chilworth, Southampton, SO16 7NP UK; 4 Institute for Naval Medicine, Crescent Road, Alverstoke, Hampshire, UK; 5 Primary Care, Population Science and Medical Education, Faculty of Medicine, University of Southampton, Highfield Campus, Southampton, UK; 6 Centre for Food Policy, City, University of London, Myddelton Street, London, UK

**Keywords:** Supermarkets, Process evaluation, Intervention implementation, Healthy eating

## Abstract

**Background:**

Placement interventions, characterised by greater availability and more prominent positioning of healthy food products in supermarkets and other food stores, are associated with healthier patterns of purchasing and diet. The WRAPPED intervention study is a natural experiment that aims to evaluate a supermarket placement intervention to improve fruit and vegetable sales, household purchasing and the dietary quality of women and their children. Process evaluation, alongside the evaluation of outcomes, is essential to understand how interventions are implemented, under what circumstances they are effective, and their mechanisms of impact. This study aimed to assess the implementation of the WRAPPED placement intervention.

**Methods:**

The study adopted a convergent mixed-methods design. Quantitative data extracted from study store planograms (visual representation of stores and product placement) before and after intervention implementation were used to assess the positioning of fresh fruit and vegetables in the first aisle from the front entrance (intervention dose). The availability of fresh fruit and vegetables in each study store was examined from stock-keeping unit (SKU) figures before and after intervention implementation. An intervention implementation survey (IIS) completed with store managers and senior supervisors before and 1- and 6-months post-intervention implementation enabled examination of the context across study stores. Semi-structured interviews with store managers and senior supervisors provided qualitative data about store staff experiences and perceptions of the intervention between 6-months post-intervention implementation.

**Results:**

The placement intervention was implemented with close adherence to the study protocol. There were marked differences, post-intervention implementation, in the positioning of fresh fruit and vegetables in intervention stores compared with control stores: median distance in intervention stores was 8.0 m (IQR 5.0 to 10.0) compared with 23.8 m (IQR 21.0 to 30.0) in control stores (*P* < 0.0001). The availability of varieties of fresh fruit and vegetables increased in intervention stores post-intervention compared with control stores: median (IQR) among intervention stores was 72 (51, 84) compared with 56.5 (50, 62) in control stores (*P* = 0.03). The mean change from baseline to post-implementation in number of different fruit and vegetables available in intervention stores was 15.3 (SD 16.7) (*P* = 0.01). IIS and interview data demonstrated little difference between intervention and store contexts over time. Reinforcing factors for intervention implementation included: head-office leadership, store staff views and attitudes and increased awareness of the importance of offering healthy food in prominent locations within stores.

**Conclusion:**

This study demonstrated that placement interventions which promote fresh fruit and vegetables to customers in discount supermarkets can be implemented effectively. These findings are encouraging for the implementation of national food policies which modify retail environments to improve population purchasing and dietary patterns.

**Trial registration:**

NCT03573973; Pre-results.

**Supplementary Information:**

The online version contains supplementary material available at 10.1186/s12966-024-01679-3.

## Background

Greater availability and more prominent positioning of healthy food products in supermarkets and other food stores is associated with healthier patterns of purchasing and diet [1, 2]. Positioning and availability are both components of placement interventions as defined by the TIPPME typology of interventions to improve population health practices [3]. The evidence of the positive effect of healthier product placement practices on consumer purchasing has influenced policy changes at national level that aim to address growing rates of obesity [4].

Improving the placement of unhealthy foods within supermarkets and large convenience stores is the focus of current UK government policy that aims to improve diet and reduce population levels of obesity [5]. The Food (Promotions and Placement) regulations [6] require large stores (> 2000 square feet), with more than 50 employees to no-longer position foods high in fat, sugar and salt (HFSS) at checkouts, aisle-ends or store entrances. The regulations came into force in October 2022 and a recent study of stakeholders demonstrated potential challenges of implementing the Food (Promotions and Placement) regulations which may undermine its effectiveness of improving population diet and obesity levels [7]. These concerns highlight the importance of understanding how supermarket interventions are implemented, particularly those that require changes to store layout and consistent compliance by store staff.

This paper describes the process evaluation of a product placement intervention within a UK supermarket chain [8]. The focus of this process evaluation was implementation of a placement intervention, being rolled out to its stores by a supermarket company, to offer insights into: (i) intervention fidelity, or consistency of intervention implementation according to the protocol, (ii) contextual factors that reinforce or work against intervention fidelity, and (iii) the environments in which placement policies are implemented.

Process evaluations which run alongside the evaluation of outcomes are essential to understand how interventions are implemented, under what circumstances they are effective, and their mechanisms of impact [9]. Assessing fidelity, which is the degree to which an intervention is implemented as originally intended [10, 11], is of particular importance in natural experiments where the real-world setting might result in adaptation of the intervention [12]. Such adaptations can moderate the relationship between the intervention and intended outcomes. Close attention should therefore be paid to assessment of what exposure to an intervention consists of by assessing a graded measure of intervention exposure and permit evaluation of dose-response relationships [13]. 

The WRAPPED intervention study is a natural experiment that aims to evaluate a supermarket placement intervention to improve fruit and vegetable sales, household fruit and vegetable purchasing and household waste, and the dietary quality of women and their children [8]. A pilot study which tested a healthier store layout in three intervention and three control stores indicated positive effects, with increased sales and purchasing of fruit and vegetables at three and six months after implementation in women shopping in intervention stores compared with those in control stores [14]. The full-scale WRAPPED study, involved 36 study stores (18 intervention and 18 control), and the intervention focused solely on fresh fruit and vegetables, by positioning an expanded fresh produce section towards the entrances of intervention stores. The results showed increased sales of fresh fruit and vegetables at the time of the intervention and 3- and 6-months after implementation, with effect sizes greater in stores where the produce section moved further forward, though reducing over time. There was a trend for a protective effect of the intervention on the proportion of families purchasing fruit and vegetables, particularly among those experiencing lower socioeconomic position. Intervention implementation was not within control of the research team.

Systematic reviews examining the implementation of supermarket interventions have demonstrated that trust and partnership between retailers and researchers, and retailers feeling they have control over the intervention, were facilitators of higher intervention fidelity [15, 16]. Lack of knowledge and control among retailers and increased demands on staff time and capacity were barriers to implementation, as was concern over intervention impacts on profit margins. Most process evaluation studies have applied qualitative assessment methods to determine in-depth insights about experiences of intervention implementation [17]. Few supermarket intervention studies have assessed intervention fidelity or dose, yet these measures are important to enable nuanced understanding of intervention effects [18]. 

This process evaluation assessed how both external and internal factors influenced intervention implementation at company and store level. The aim of this study was to assess the level of implementation of the WRAPPED full-scale in-store intervention and factors that may have reinforced or driven deviations from the research protocol [8]. The study adopted a convergent mixed-method approach incorporating objective quantitative data on fidelity and intervention dose, alongside qualitative data about the experiences and perceptions of store staff in intervention and control stores [19]. The two specific research questions (RQ) addressed in this study include: (i) RQ1- How closely did intervention implementation adhere to the study protocol?; and (ii) RQ2 - What contextual factors affected intervention implementation?

## Methods

### Setting and sample

**Fig. 1:**
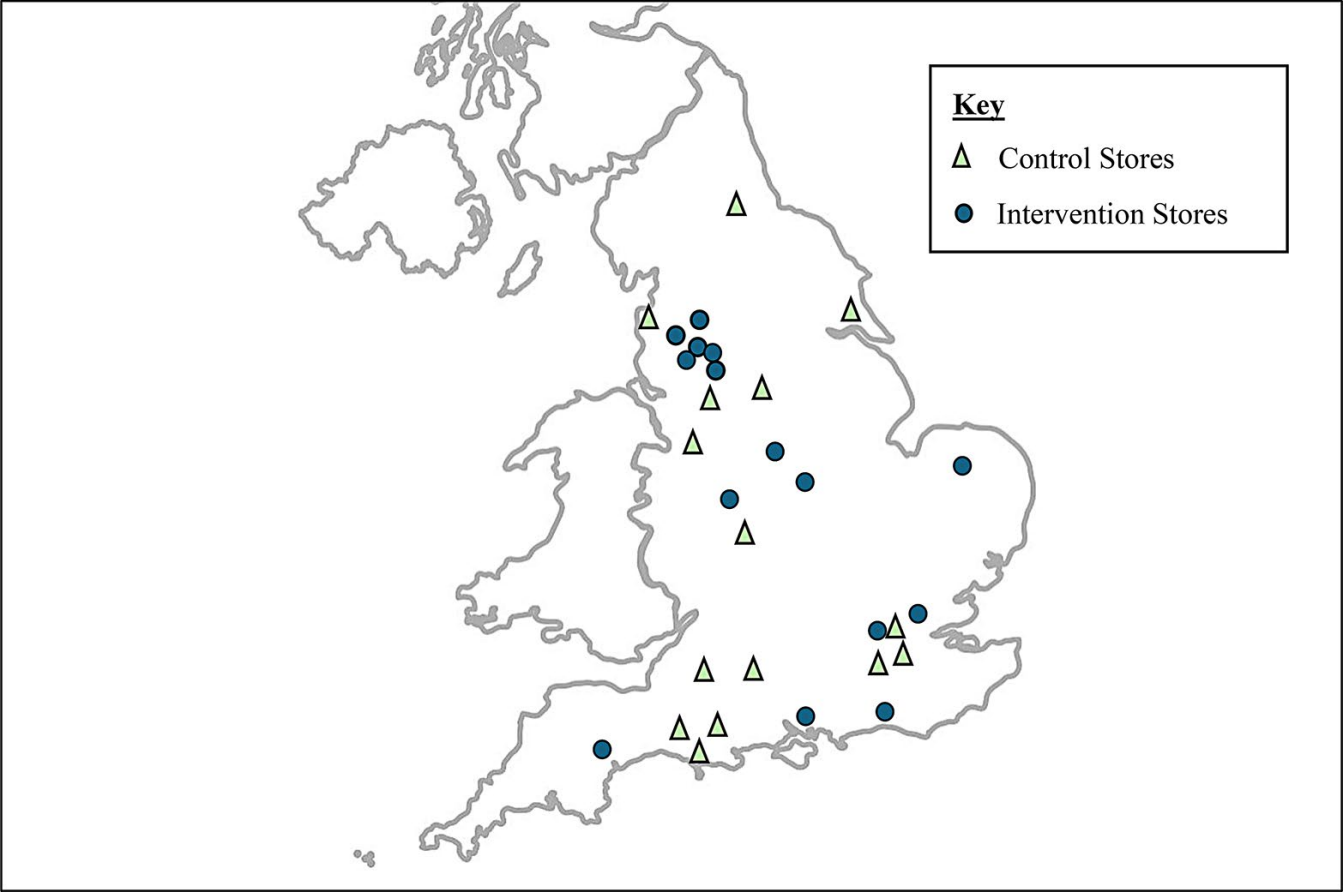
Geographical location of participating supermarkets

The setting for this study was a national UK discount supermarket chain in which a natural experiment was undertaken which enhanced the placement of fresh fruit and vegetables by relocating an expanded fresh produce section towards the front of the store [8]. A prospective matched controlled cluster trial was used to assess the effects of the intervention on store sales, customer purchasing, dietary quality of women and their young children, and household fruit and vegetable waste. Participants were recruited from 36 study stores (*n* = 18 intervention and *n* = 18 matched control stores) located in England, UK (Fig. 1). The process evaluation described in this paper ran concurrently to the evaluation of outcomes from March 2018 to May 2022.

### Data collection

This process evaluation used a convergent mixed-method approach [19, 20] to assess implementation and consisted of four components resulting in four data sources that were used to address the two research questions. These four data sources include:

1. Quantitative data extracted from study store planograms (visual representation of stores and product placement) before and after intervention implementation to assess the positioning of fresh fruit and vegetables and other products in the first aisle from the front entrance.
2. Quantitative data on the availability of fresh fruit and vegetables in each study store from stock-keeping unit (SKU) figures before and after intervention implementation.
3. Quantitative data about the context within intervention and control stores collected via an intervention implementation survey (IIS) completed with store managers or senior supervisors before and 1- and 6-months post-intervention implementation.
4. Qualitative data about store staff experiences and perceptions of the research study collected via semi-structured interviews with store managers and senior supervisors between 6- to 12-months post-intervention.

Table 1 shows the timing of data collection and how the four datasets were used to answer each of the research questions.

**Table 1:** Research questions and data sources

Research question (RQ)	Data source	Timing of data collection	Variables measured
RQ1: How closely did intervention implementation adhere to the study protocol?	Planograms before and after intervention, provided by the collaborating supermarket	Baseline and after implementation of the placement intervention*	Distance in metres (m) fresh fruit and vegetable section moved forward
	Store-level SKU data on available produce, provided by collaborating supermarket	Baseline and after implementation*	Availability of fresh fruit and vegetables – number of varieties of fresh fruit and vegetables
	Intervention implementation survey	Baseline, 1 month and 6 months after intervention implementation	Proportion of stores with different products available in the first half of the first aisle
RQ2: What contextual factors affect intervention implementation?	Intervention implementation survey	Baseline, 1 month and 6 months after intervention implementation	Proportion of stores with produce placed prominent locations (foyer, checkouts, aisle-ends) Regularity of deliveries of fresh fruit and vegetables
	Semi-structured interviews with store managers and senior store staff	Post-implementation, for most stores these took place at least one year after implementation	Study store manager views and lived experience of implementing the intervention

* at the time of store refit

#### RQ1: how closely did intervention implementation adhere to the study protocol?

**Fig. 2:**
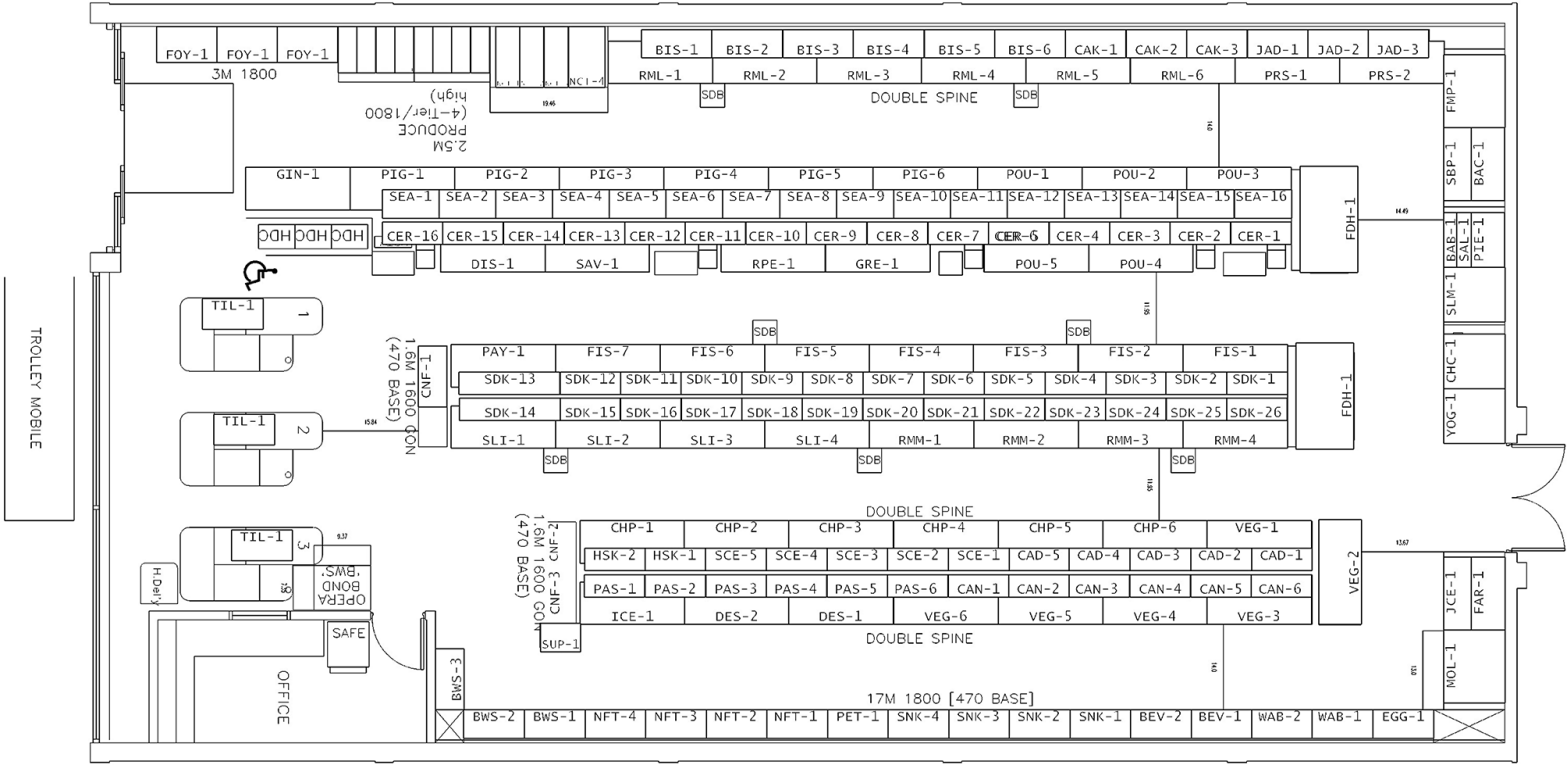
Example of a store planogram

Intervention implementation and intervention dose were assessed through analysis of the three quantitative datasets (planogram, SKU and IIS data) (Table 1). Change in fruit and vegetable positioning from baseline to post-implementation was assessed by comparing planograms before and after implementation in intervention and control stores and provided the measure of intervention dose for fruit and vegetable section positioning. Figure 2 shows an example of a store planogram which is a visual representation of stores, outlining specific positioning of product categories.

These planograms were generally updated every 4–12 weeks. The size of some display units (such as freezers) was known and consistent across study stores and the lengths of displays were marked on the planograms. Using these measures, it was possible to estimate the distance that the produce section moved within each intervention store. One researcher (HP) estimated the distances and discussed estimates with two team members (SC, CV) to reach consensus. The distance of fresh fruit and vegetables from the store entrance (metres from first products placed at store entrance) at baseline and post-intervention implementation were calculated for all stores and the change in metres the produce section moved forward deduced for intervention stores. Baseline planograms could not be obtained for eight of the intervention stores. In these instances, missing baseline information on distance of the fruit and vegetable section from the entrance was obtained from photographs researchers had taken within stores while conducting in-store recruitment during the baseline period.

The measure of intervention dose for availability of fresh fruit and vegetables offered before and after intervention implementation was examined using SKU data. Numerical SKU data for each study store at baseline and immediately post-intervention implementation were provided by the head-office team of the collaborating supermarket. The planogram and SKU data were provided by the supermarket chain at the time of store refits. Data from the IIS provided longer term measures of product positioning and availability.

The IIS was designed specifically for this study to collect data about the context within each study store. This contextual data enabled assessment of intervention fidelity and other in-store contextual factors which may have impacted intervention effects. The IIS was developed by the research team who have expertise in retail food environment research and process evaluation (CV, JB, SC) [18, 21, 22]. Specific items in the IIS included: (i) the range of products located near the store entrance or ‘foyer focus’ (where branded products are often positioned) in the past month, (ii) the range of products positioned in the front half of the first aisle in the past month, (iii) the range of products positioned at checkouts and aisle-ends opposite checkouts in the past month, (iv) the regularity of delivery of fruit and vegetables in the past month, and (v) whether health-related promotions had occurred in the store in the past month. The survey was completed in-person at baseline through discussion with store managers and senior staff, and observation within stores. At 1- and 6-months post-implementation, the survey interview was completed by phone interview. Store visits and phone calls were pre-arranged via the collaborating supermarket’s head-office. Median (IQR) days for survey completion after the specified time point were 3 (-1, 10), 23 (18, 34) and 12 (-5, 26) for baseline, and 1- and 6-months follow-up respectively.

#### RQ2: what contextual factors affected intervention implementation?

Assessing the factors that might explain variability in intervention implementation was addressed using two data sources (Table 1): (i) the IIS, described above; and (ii) semi-structured interviews with study store managers and senior staff. These interviews were completed with 30 store managers and senior supervisors between October 2020 and February 2022. This time period corresponded with 6- to 12-months post-intervention implementation. The collaborating supermarket facilitated contact with store staff for these interviews. An email invitation was sent to store managers by a member of the research team and a follow-up phone call was made to the store to book a convenient time for staff to complete the interviews. Staff were assured of anonymity of their responses and consent forms were completed by phone prior to the interview. The interview guide was developed and tested in the WRAPPED pilot study [14] and then refined in preparation for the full-scale study [8]. The interview guide (Supplementary Table 1) included questions about (i) staff experiences of being involved in the study (ii) the perceived influence of placement strategies on customers’ choices and (iii) the drivers and challenges for retailers in providing more healthful retail environments to customers. Staff were informed that the purpose of the interview was to understand their views and experiences of the intervention and the perceived impact of product placement on customers. The semi-structured nature of the interviews allowed exploration of topics of interest in a broad, systematic manner whilst also permitting store managers to discuss their lived experiences of implementing the intervention and raise points important to them [23]. The original sample size target was to interview 24 staff (12 from intervention and 12 from control stores) [8]. Recruitment of study store staff extended beyond this figure because new themes continued to emerge that provided information to address the research questions. Questions were not provided to participants in advance of the interview. All interviews were conducted by PD and lasted between 10 and 28 min. All interviews were audio-recorded and transcribed verbatim, eliminating any personal or identifiable details. QSR NVIVO Software 12 was used to organise and analyse the data.

### Data analysis

Summary statistics were produced, presented as mean (SD) for normally distributed continuous variables and median (IQR) for non-normally distributed continuous variables. Categorical variables were calculated as n (%). Mann-Whitney U-tests were used to compare non-normally distributed variables between intervention and control stores, whilst two-sample t-tests were used to compare normally distributed variables between intervention and control stores. One-sample t-tests were used to assess whether normally distributed changes in intervention stores were different to zero. Fisher’s exact tests and chi-squared tests were used to compare categorial variables between intervention and control stores (Fisher’s exact tests were used over chi-squared tests when more than 20% of the cells had expected frequencies less than five) [24]. All analyses were performed in Stata 14 [25]. 

Qualitative data were analysed using inductive reflexive thematic analysis following Braun and Clarke’s guidelines to ensure that themes and subthemes were derived from the raw data [26]. Two researchers (PD, HP) read and familiarised themselves with the data and identified initial codes. Codes were then organised into themes and subthemes to develop an initial framework, which was then refined through further coding of each transcript. A selection of transcripts was double coded to further refine the coding framework (WL, CV). The coding framework was then finalised, and validity of themes discussed by PD, CV, JB and WL. Each transcript was then recoded to the final coding framework. The relativist ontological and subjective epistemic position approach was taken for this analysis which states that reality is a matter of individual perspective and based on personal experience and insight [27]. Themes and sub-themes were compiled together with verbatim quotes. The analysis was conducted in a manner that considered differences between staff from intervention and control stores.

Team members (PD, CV, JB, WL) were women aged 30–63 years, with ethnic representation and expertise in public health nutrition, food policy, evaluation of complex interventions, and psychology.

### Ethics and governance

Ethical approval for the study was given by University of Southampton Faculty of Medicine ethics committee (Ethics ID 20986.A9). The study abided by the Declaration of Helsinki, Research Governance Framework for Health and Social Care and Data Protection regulations. The Consolidated criteria for Reporting Qualitative research (COREQ) were used to report qualitative research methods used in this study [28]. 

## Results

### Store and participant samples

Planogram data were obtained for all 36 study stores (18 intervention and 18 control), confirming the location of fresh fruit and vegetable sections post-intervention implementation.

SKU data which provided the number of varieties of fresh fruits and vegetables available in study stores were available for 29 stores before the intervention and all stores after the intervention.

The IIS was completed for 35 of the 36 study stores (Table 2). Disruptions caused by the covid-19 pandemic prohibition completion of the IIS at each time point for all stores. Surveys were not completed by three stores at baseline, 12 at 1-month follow-up and one at 6-month follow-up.

**Table 2:** Frequency of stores which completed intervention implementation surveys (IIS) at baseline, 1- and 6-months post-implementation

*n (%) stores*			
Time point	Control	Intervention	Total
Baseline	16 (44%)	17 (47%)	33 (91%)
1 month	13 (36%)	11 (31%)	24 (67%)
6 months	18 (50%)	17 (47%)	35 (97%)

A total of 30 supermarket staff (15 intervention stores, 15 control stores) completed semi-structured interviews between October 2020 and February 2022. More than three quarters of staff who completed these interviews held the position of store manager (Table 3).

**Table 3:** Semi-structured interview participant characteristics

Store group	*n* (%)	Staff position	*n* (%)
Control stores	15 (50)	Store Manager	11 (37)
		Senior Supervisor	4 (13)
Intervention stores	15 (50)	Store Manager	12 (40)
		Senior Supervisor	3 (10)

### RQ1: how closely did intervention implementation adhere to the study protocol?

#### Distance of fresh fruit and vegetable section from store entrance

Planogram data showed that the distance of fresh fruit and vegetables from the store entrance was markedly shorter in intervention compared to control stores after intervention implementation. The median distance for intervention stores was 8.0 m (IQR 5.0 to 10.0) compared with 23.8 m (IQR 21.0 to 30.0) for control stores (*p* < 0.0001). The mean distance that the fresh fruit and vegetable section moved forwards in intervention stores from baseline to post-implementation was 14 m (SD 9.7) (*p* < 0.0001).

#### Availability of fresh fruit and vegetables

Analysis of SKU data showed that the mean number of different fruits and vegetable products available was higher in intervention compared with control stores post-intervention implementation. The median (IQR) value among intervention stores was 72 (51, 84) compared with 56.5 (50, 62) among control stores (*p* = 0.03). The mean change from baseline to post-intervention implementation in number of different fruit and vegetables available in intervention stores was 15.3 (SD 16.7) (*p* = 0.01).

#### Positioning of fresh fruit and vegetable section

IIS data (Table 4) showed only small differences between the intervention and control stores at baseline in the positioning of fresh fruit and vegetables in the front half of the first aisle in the month preceding implementation. Among control stores 27% indicated fresh produce in this in-store location compared to 41% of intervention stores (*p* = 0.39). At 1-month post-intervention implementation, fresh fruit and vegetables were positioned in the front half of the first aisle in all intervention stores (100%) compared with less than a quarter (23%) of control stores (*p* < 0.001). At 6-months post-implementation, 94% of intervention stores reported fresh produce in the front half of the first aisle compared to 39% of control stores (*p* = 0.001). There were no significant differences between intervention and control stores in the positioning of other products included in the questionnaire in the front half of the first aisle, including for frozen fruit and vegetables (all *p* > 0.10).

**Table 4:** Number (proportion) of stores with listed products located in front half of the first aisle in month preceding the intervention implementation survey

Outcome	Baseline				1 month				6 months			
	Control	Inter-vention	*P*-value*	*n*	Control	Inter-vention	*P*-value*	*n*	Control	Inter-vention	*P*-value*	*n*
Slimming World (ready meals)	8 (50%)	7 (41%)	0.61	33	3 (23%)	0 (0%)	0.22	24	8 (44%)	4 (25%)	0.24	34
Weight Watchers	0 (0%)	1 (8%)	0.48	25	0 (0%)	0 (0%)	1.00	23	0 (0%)	0 (0%)	1.00	25
Natural frozen fish fillets	11 (69%)	10 (59%)	0.55	33	6 (46%)	1 (9%)	0.08	24	8 (44%)	3 (19%)	0.11	34
Breaded/battered frozen fish products	10 (63%)	11 (65%)	0.90	33	6 (46%)	2 (18%)	0.21	24	9 (50%)	5 (31%)	0.27	34
Raw frozen meat	3 (19%)	6 (35%)	0.29	33	0 (0%)	1 (9%)	0.46	24	4 (22%)	5 (29%)	0.71	35
Value-added meat (kebabs, meatballs)	9 (56%)	8 (47%)	0.60	33	3 (23%)	2 (18%)	1.00	24	5 (28%)	6 (38%)	0.55	34
Raw frozen poultry	7 (44%)	8 (47%)	0.85	33	5 (38%)	2 (18%)	0.39	24	10 (56%)	5 (31%)	0.15	34
Value-added poultry (picked eggs, crepes)	5 (31%)	10 (59%)	0.11	33	4 (31%)	3 (27%)	1.00	24	12 (67%)	8 (50%)	0.32	34
Pizza	4 (25%)	4 (24%)	1.00	33	7 (54%)	4 (36%)	0.39	24	3 (17%)	3 (19%)	1.00	34
Party food	1 (6%)	2 (12%)	1.00	33	2 (15%)	0 (0%)	0.48	24	2 (11%)	4 (24%)	0.40	35
Seasonal confectionary	9 (56%)	7 (41%)	0.39	33	6 (46%)	8 (73%)	0.24	24	11 (61%)	14 (88%)	0.13	34
Standard confectionary	5 (31%)	2 (12%)	0.23	33	3 (23%)	0 (0%)	0.22	34	7 (39%)	4 (25%)	0.39	34
Fresh fruit and vegetables	4 (27%)	7 (41%)	0.39	32	3 (23%)	11 (100%)	< 0.001	24	7 (39%)	16 (94%)	0.001	35
Frozen fruit and vegetables	0 (0%)	1 (6%)	1.00	33	0 (0%)	0 (0%)	1.00	24	0 (0%)	1 (6%)	0.49	35
Christmas trimmings	0 (0%)	1 (6%)	1.00	33	0 (0%)	3 (30%)	0.08	22	1 (6%)	2 (12%)	0.60	35
Christmas meat	0 (0%)	0 (0%)	1.00	33	0 (0%)	2 (20%)	0.20	22	1 (6%)	2 (12%)	1.00	34

*P-values compare control and intervention at each time point. P-values are chi-squared except where data are too sparse (fewer than 80% of cells have an expected frequency greater than five, or not all cells have an expected frequency greater than one) in which case a two-sided Fisher’s exact test is used

^a^ Slimming World ready meals are portion-controlled frozen options intended to support weight management by being lower in calories and fat, while offering a combination of ingredients designed to provide satiety and a variety of flavours

### RQ2: what contextual factors affected intervention implementation?

IIS data were used to compare differences in store context between intervention and control stores and to examine changes in store context over the study period. These data showed small non-significant differences between intervention and control stores in the positioning of less healthy food products at store entrances in the foyer focus across the three time points (all *p* > 0.10) (Table 5). There were also few changes to intervention stores in the positioning of less healthy produce during the follow-up period. Similar patterns of product positioning in intervention and control stores were observed for checkouts and aisles-ends opposite checkouts, with little change over time or between groups (all *p* > 0.30) (Supplementary Tables 2 and 3).

**Table 5:** Number (proportion) of stores with listed products located in the foyer focus (front of the store area) in month preceding the intervention implementation survey

Outcome	Baseline				1 month				6 months			
	Control	Inter-vention	*P*-value*	*n*	Control	Inter-vention	*P*-value*	*n*	Control	Inter-vention	*P*-value*	*n*
Slimming World (ready meals)^a^	8 (50%)	7 (41%)	0.61	33	3 (23%)	0 (0%)	0.22	24	8 (44%)	4 (25%)	0.24	34
Weight Watchers	0 (0%)	1 (8%)	0.48	25	0 (0%)	0 (0%)	1.00	23	0 (0%)	0 (0%)	1.00	25
Natural frozen fish fillets	11 (69%)	10 (59%)	0.55	33	6 (46%)	1 (9%)	0.08	24	8 (44%)	3 (19%)	0.11	34
Breaded/battered frozen fish products	10 (63%)	11 (65%)	0.90	33	6 (46%)	2 (18%)	0.21	24	9 (50%)	5 (31%)	0.27	34
Raw frozen meat	3 (19%)	6 (35%)	0.29	33	0 (0%)	1 (9%)	0.46	24	4 (22%)	5 (29%)	0.71	35
Value-added meat (kebabs, meatballs)	9 (56%)	8 (47%)	0.60	33	3 (23%)	2 (18%)	1.00	24	5 (28%)	6 (38%)	0.55	34
Raw frozen poultry	7 (44%)	8 (47%)	0.85	33	5 (38%)	2 (18%)	0.39	24	10 (56%)	5 (31%)	0.15	34
Value-added poultry (picked eggs, crepes)	5 (31%)	10 (59%)	0.11	33	4 (31%)	3 (27%)	1.00	24	12 (67%)	8 (50%)	0.32	34
Pizza	4 (25%)	4 (24%)	1.00	33	7 (54%)	4 (36%)	0.39	24	3 (17%)	3 (19%)	1.00	34
Party food	1 (6%)	2 (12%)	1.00	33	2 (15%)	0 (0%)	0.48	24	2 (11%)	4 (24%)	0.40	35
Seasonal confectionary	9 (56%)	7 (41%)	0.39	33	6 (46%)	8 (73%)	0.24	24	11 (61%)	14 (88%)	0.13	34
Standard confectionary	5 (31%)	2 (12%)	0.23	33	3 (23%)	0 (0%)	0.22	34	7 (39%)	4 (25%)	0.39	34
Fresh fruit and vegetables	4 (27%)	7 (41%)	0.39	32	3 (23%)	11 (100%)	< 0.001	24	7 (39%)	16 (94%)	0.001	35
Frozen fruit and vegetables	0 (0%)	1 (6%)	1.00	33	0 (0%)	0 (0%)	1.00	24	0 (0%)	1 (6%)	0.49	35
Christmas trimmings	0 (0%)	1 (6%)	1.00	33	0 (0%)	3 (30%)	0.08	22	1 (6%)	2 (12%)	0.60	35
Christmas meat	0 (0%)	0 (0%)	1.00	33	0 (0%)	2 (20%)	0.20	22	1 (6%)	2 (12%)	1.00	34

*P-values compare control and intervention at each time point. P-values are chi-squared except where data are too sparse (fewer than 80% of cells have an expected frequency greater than five, or not all cells have an expected frequency greater than one) in which case a two-sided Fisher’s exact test is used

^a^ Slimming World ready meals are portion-controlled frozen options intended to support weight management by being lower in calories and fat, while offering a combination of ingredients designed to provide satiety and a variety of flavour

IIS data showed that the proportion of stores offering health-related food promotions did not differ between intervention and control stores at baseline (*p* = 0.26), 1-month (*p* = 0.42) or 6-months follow-up (*p* = 0.10) (Supplementary Table 4). There were also no differences in the regularity of fresh fruit and vegetable deliveries. All stores (control and intervention) received regular deliveries of fruit and vegetables at all time points.

Results from the qualitative analysis of the 30 semi-structured interviews identified three themes from store managers and senior supervisors that help explain deviation from or adherence to the intervention protocol in relation to the positioning and availability of fruit and vegetables. These interviews also enabled examination of contextual factors in stores at the time of intervention, particularly in relation to placement of unhealthy products. The three key themes that emerged from the analyses are outlined below with illustrative quotes from participants.

#### Theme 1: the supermarket chain’s head-office supported implementation of the intervention

Store managers and senior supervisors described the way in which processes across the company supported the implementation of the intervention. Head office was responsible for setting the planogram structure which determined the store layout and positioning of products, and store managers and staff were responsible for implementing these within stores. This process was also followed for the fruit and vegetable section of the intervention.

*The layouts come down on our computers systems and we just picture and print them and implement them.* 5183 Intervention, store manager.

*Our fruit and veg is now up at the front of the shop*,* whereas it wasn’t before. So*,* we have a bigger section than what we did before.* 983 Intervention, store manager.

Participants reported that the additional supply of fresh fruit and vegetables needed for intervention implementation were readily available within stores. Supply chains kept up the additional demand and regular deliveries.

*They keep supplying us with enough stock to replenish it so it mustn’t be an issue at the moment with displays.* 5111 Intervention, senior supervisor.

*So*,* actually*,* [supermarket chain] has placed quite a lot of emphasis on produce over the past 12 weeks. So*,* I can’t really say there’s been a supply issue*,* we’ve had strawberries as well*,* that’s on the shop floor so no I wouldn’t say it’s a supply issue in terms of produce.* 5183 Intervention, store manager.

It was evident across both intervention and control stores that staff felt the role of the supermarket chain was important in influencing customer choice. The placement of products within stores contributed to this influence and government was increasingly interested in promoting retailers to support healthy choices.

*If the government asks supermarkets to trade a lot more on healthier products than unhealthy products*,* then obviously that’s certainly the way forward that retailers will probably end up going.* 2903 Intervention, store manager.

*So as a food retailer you got to find a healthy balance*,* but in line with obviously what the government are trying to do in trying to steer people towards living a healthier lifestyle. As a food retailer you’ve got to support that and kind of play your part*,* and it can be very difficult because as a company we do what we know works and that’s flog the unhealthy options.* 4835 Control, store manager.

#### Theme 2: at the store level managers have a degree of autonomy to position healthy and unhealthy products to promote sales

Store managers and senior supervisors reported that they were able to make decisions about the positioning of baskets, boxes and bins which are not fixed infrastructure. They described how they drew on their knowledge of the store and their customers’ preferences to make decisions about the types products placed in these moveable display units. The items in display bins or baskets were often less healthy and prominently positioned around the store.

*The first aisle is the company. The front of the store is mine. So*,* the checkout area is mine*,* and it’s up to me. I have to make the money from that point. And know what the public will tell us*,* good for them*,* good for me.* 3367 Intervention, store manager.

*Head office decide what products go where in each store. So*,* anything that’s an additional purchase*,* so whether it’s in a bin or on a stack*,* then we can select those additional lines. But any core range*,* whether it’s on a shelf or in a freezer*,* fridge*,* that’s all dealt with at head office.* 2467 Intervention, store manager.

*And then there are trolley bays at the front which are company-specific layouts*,* it’s very rare that there’s something that’s a healthy product on there. So those bays at the front are by the buyers and the merchandisers. And then we have a selected list that we can choose lines from. Everything from the store manager’s point of view is very rigid from the moment the customer walks in the door*,* until they get to the end of the first aisle. Then we have the autonomy to do anything as store managers.* 3881 Control, senior supervisor.

There was evidence that this autonomy for store managers could have led to beneficial changes in prominent positioning of healthy products in control stores.

*It [WRAPPED study] made us think more about where we’re putting the healthier options within the store and we do place more [fresh produce] within the first aisle or within the entrance now.”* 5543 Control, store manager.

*It [WRAPPED study] has helped us pick our conscience or prick our minds to think as to what put and where because if say we have a Slimming World cabinet we wouldn’t put chocolate in front of it whereas before we might have. I would say as a whole maybe to get managers/supervisors to think about where they’re putting stacks and what they’re putting there you know.”* 291 Control, store manager.

Participants reported that implementing the WRAPPED intervention had no impact on the workload of staff at store level. This finding indicates that store managers did not notice a drain on staff resource and could successfully manage differences in staff time or effort within existing resources.

*No*,* the study itself didn’t change my workload.* 2467 Intervention, store manager.

*So if we set up right in the morning and we check the good quality and check the good dates*,* that actually makes our workload easier throughout the day. You just have to go out and tidy it up once a day*,* you don’t really have to fill it too much now because of all the additional space that we’ve got now it helps to keep it on sale for longer.* 3827 Intervention, store manager.

#### Theme 3: implementation of the intervention had a positive impact on store staff that further reinforced fidelity

It was clear that store managers and senior supervisors believed that the prominent positioning and increased availability of fresh fruit and vegetables had boosted store sales. This impact was felt to be positive for the store and their customers.

*I think most stores that have had a refit that have got the produce down the front it does sell better. I used to work in another store*,* and before the refit ours was right at the bottom and then once it got moved to the front*,* I think the sales was up by 25%*,* on produce just by moving it to the front.* 411 Intervention, store manager.

*Obviously we’ve got all our produce and bananas and stuff at the front of the store*,* and they sell more now than they ever did.* 5111 Intervention, senior supervisor.

*It’s a noticeable difference that*,* for instance*,* fruit and veg in the shop sells well because it’s down the first aisle*,* and in other stores it’s pushed towards the back of the shop.* 2903 Intervention, store manager.

*I think with the healthy options*,* having your produce down the first aisle makes a massive difference. Like people do tend to pick up their fruit and veg.-* 1411 Intervention, store manager.

*It definitely does determine a customer to buy more because I mean for example we’ve got water*,* at the very beginning of our store now*,* we’ve got water*,* bananas*,* tangerines*,* like I say*,* carrots and I’ve sold loads of them this week. So it does make a massive impact.* 5395 Intervention, senior supervisor.

Store managers and senior supervisors reported how the intervention had positively impacted on their own views towards healthy eating. They reported noticing similar changes among their colleagues and on their customers attitudes.

*Well*,* it made us more aware of healthy eating and product placement in general. And the way that items are merchandised and the way that the customer has that choice to sort of have the healthy options however also have other options. And obviously it’s increased awareness throughout the team. The study made us more aware of healthy eating basically.* 5395 Intervention, senior supervisor.

*It makes them (customers) think about it a bit more*,* you know it certainly puts it in their eye line and then makes some consider it before they get to the tried and tested method of a chocolate bar or a biscuit.* 3287 Intervention, store manager.

## Discussion

### Summary of findings

This process evaluation showed that implementation of the placement strategy in our large supermarket intervention trial closely adhered to the protocol, showing high fidelity for both intervention components: more prominent positioning and increased availability of fresh fruit and vegetables. The process evaluation data, however, revealed differences in intervention dose across study stores both for distance the produce section moved forward toward the store entrance and the number of fresh fruit and vegetable items available. No other discernible in-store differences were observed between intervention and control stores.

Semi-structured interviews with store staff showed leadership for the intervention from the supermarket chain’s head-office through provision of store planograms to enhance protocol adherence and robust supply chain management ensuring regular deliveries of fresh produce. Staff also acknowledged the autonomy of store managers and senior staff in positioning mobile product display units to increase sales and that this activity may have increased the availability and prominent positioning of unhealthy foods in intervention stores and/or promotion of fruit and vegetables near store entrances in control stores. Staff also described several store-level factors which positively reinforced intervention implementation including: (i) the increased sales of fresh fruits and vegetables observed following intervention implementation; (ii) heightened staff awareness of the powerful role positioning items in prominent locations has on prompting customers’ choices and (iii) staff recognition that supermarkets play an important role in supporting healthier diets when implemented collectively.

### Comparison with existing research

This study provides nuanced insights about the dose of implementation for the product placement intervention under examination and has extended methods used in previous literature through the use of store planograms for positioning calculations. Measures of intervention dose are important to enable nuanced understanding of intervention effects which has been rarely undertaken in healthy product placement research [18, 29] and its value is demonstrated in the primary outcome evaluation for the WRAPPED study which revealed a greater effect size among intervention stores where the produce section moved a greater distance towards store entrances. A Cochrane review of availability and positioning trials in laboratory settings and food service settings indicated similar dose-relationships for unhealthy foods [30]. Assessment of intervention dose is particularly important for interpreting the effects of natural experiments on outcomes of interest.

The important role of the supermarket chain’s head-office in achieving successful intervention implementation which were revealed in this study from the qualitative interviews with store staff are consistent previous research in this field. Store managers and senior supervisors indicated that a strong commitment for the intervention from the supermarket chain’s head office and highlighted their own compliance with these instructions from head office. In a recent evaluation of a food retail intervention to reduce unhealthy purchases in remote areas of Australia, organisational commitment through leadership and provision of resources to support implementation, were reported as being instrumental to effective implementation [31]. While the organisation implementing the intervention in the Australian study was the not the supermarket chain itself, but rather a not-for-profit private company, it was similar to the supermarket chain in our study in owning or managing stores. Systematic reviews investigating factors influencing implementation of healthy food retail interventions also pointed to the importance of the organisational values of retailers, particularly their attitudes to the communities they served, in supporting implementation [15, 17]. Additionally, having control over intervention implementation was considered a reinforcing factor for fidelity across the literature [16, 32]. The natural experiment design of the current afforded the supermarket chain full control over intervention implementation which likely supported the positive contextual factors observed in this study including assurance of supply chains for fresh produce, minimising inconsistencies in the retail environments between intervention and control stores and reducing burden on store staff.

Store managers have been identified in previous research as being key facilitators in achieving successful implementation of healthy food retail interventions, particularly when the intervention aligns with their personal beliefs and values [15, 17]. Our findings show that the increased sales of fruit and vegetables and positive feedback from staff and customers reinforced intervention implementation, likely due to the alignment with their personal values. The Australian study described above similarly found that store managers’ beliefs in the benefits of the intervention for their customers was pivotal to implementation. Store managers can, however, also hinder intervention implementation particularly when concerns about the conflict between commercial and health benefits arise [17]. This intervention described in the current study offers mutual benefit by increasing the prominence and sales of healthy foods, although intervention store staff did mention using mobile displays of unhealthy products in prominent first aisle locations to boost overall store sales.

### Strengths and limitations

This study used methods that were consistent with guidance on best practice in process evaluation [9] and provides novel insights for the implementation of placement interventions in retail outlets, particularly in relation to assessment of intervention dose and factors affecting the fidelity of product placement strategies. We adopted a convergent mixed-method approach [20] to maximise understanding of intervention fidelity, intervention dose and contextual drivers of intervention implementation. Quantitative data were collected to assess implementation of the intervention, with objective data in the form of distances, in metres, of the positioning of produce from the front of the study store. It was intended that planogram data would be confirmed after intervention implementation by members of the research team through photos and observations. Store visits, however, were not feasible during the pandemic due to COVID-related restrictions, severely limiting the number of stores for which we obtained these data. The responses from store managers to intervention implementation surveys provided additional confirmation of the planogram data. Additional in-store information regarding shelf-positioning, signage strategies (posters, branded shelving etc.) or additional marketing such as use of mobile products displays would have provided additional useful information about in-store marketing practices of both healthy and unhealthy foods.

Quantitative data were complemented by qualitative data from semi-structured interviews with store managers and senior staff to provide deeper understanding of factors related to the research questions. These different sources of data helped to minimise bias that may have arisen from reliance on a single data source and provided a detailed picture of the factors influencing intervention implementation.

It was not possible to conduct surveys with all 36 study stores (18 were intervention and 18 control stores) at all time points due to the impact of the covid-19 pandemic on store manager workloads. Nevertheless, all but two intervention stores provided data at baseline and all but one took part in the final 6-month follow-up survey; figures were equivalent for control stores. The baseline and 6-month time-points are particularly important for the WRAPPED primary outcome evaluation. The self-reported nature of the survey responses may introduce some bias to the results. However, methods were applied consistently across intervention and control stores minimising impacts on final analyses. All semi-structured interviews were conducted with senior store staff from a discount supermarket chain. It is possible that more junior store staff or staff from high-range or other chain supermarkets would provide different views which may reduce generalisability of the qualitative findings. None-the-less the original sample size for the qualitative interviews was extended because new findings continued to emerge, indicating the breadth of views covered in this dataset.

### Implications for policy and practice

Process evaluations of complex natural experiments, like the WRAPPED study, are vital to provide value insights into the circumstances in which they are effective. This evaluation of the implementation of the WRAPPED product placement intervention has particular relevance for current UK food policy by providing important information about the environments within which product placement strategies are implemented. A key finding which holds direct relevance for the UK Food (Promotions and Placement) regulation [6] is the revelations from store staff about the autonomy they hold to make decisions about the positioning of mobile product display units. This promotional strategy is used by store managers and senior store staff to boost sales based on their knowledge of their own customers. Such practices are often prompted by head-office directed competition between stores within a geographical region and frequently involve prominent positioning of unhealthy foods to boost sales. directed competition between stores within a geographical region and frequently involves prominent positioning of unhealthy foods to boost sales. The findings of this study showed there was no change in the positioning of unhealthy products in prominent locations despite the introduction of fresh fruit and vegetables near the front of stores. This finding suggests that fruit and vegetables were co-located with unhealthy food and highlights the need for regulations that limit prominent positioning of unhealthy items while also ensuring prominent positioning of healthy foods, such as fruit and vegetables, to avoid sending mixed messages to consumers.

Supermarket chain commitment to the implementation of healthy food interventions and policies is vital for leadership and resource provision. But store managers role in overseeing implementation is pivotal and further research of this understudied area is needed to ensure effective and implementation of healthy food retail policies [7]. 

Involvement in this study prompted an open acknowledgement from senior store staff of a positive change in their attitudes about the important role of supermarkets in promoting healthier food choices to customers. These findings are encouraging and suggest increasing support from the supermarket sector for further food policies which enable collective action across retailers.

## Conclusion

A large placement intervention trial to increase the availability and prominent positioning of fresh fruit and vegetables in discount supermarkets was successfully implemented across intervention stores. Using a convergent mixed-methods approach to evaluate intervention implementation can be useful at identifying the dose of intervention implementation and contextual factors driving adherence to or divergence from the protocol. Involvement in healthy retail interventions can alter store staff attitudes about the role of supermarkets in promoting healthy choices to consumers and in achieving successful implementation.

## Electronic supplementary material

Below is the link to the electronic supplementary material.

Supplementary Material 1

Supplementary Material 2

Supplementary Material 3

## Data Availability

Data described in this manuscript that has been collected by the research team during this study, and that which can be anonymized, will be made available upon reasonable request to the data manager (Vanessa Cox, vac@mrc.soton.ac.uk) pending their approval. Purchasing and sales data are confidential and will not be made availability because of the conditions of the agreement with the commercial collaborator.
