# Supplementary material for: Implementation of a UK supermarket intervention to increase purchasing of fresh fruit and vegetables: process evaluation of the WRAPPED natural experiment
Source: Int J Behav Nutr Phys Act. 2024 Nov 11;21:128. doi: 10.1186/s12966-024-01679-3 (PMC11552182; doi:10.1186/s12966-024-01679-3)
Supplement: Supplementary file 2 — Supplementary Material 2 [file 12966_2024_1679_MOESM2_ESM.doc]

STROBE Statement—Checklist of items that should be included in reports of ***cross-sectional studies***

|  | Item No | Recommendation |
| --- | --- | --- |
| **Title and abstract** | 1 | (*a*) Indicate the study’s design with a commonly used term in the title or the abstract – title indicates that the study is a process evaluation |
|  |  | (*b*) Provide in the abstract an informative and balanced summary of what was done and what was found – yes, see page 3 |
| Introduction | | |
| Background/rationale | 2 | Explain the scientific background and rationale for the investigation being reported – see page 5 to 7 |
| Objectives | 3 | State specific objectives, including any prespecified hypotheses – the research questions are given on page 7 at the end of the introduction |
| Methods | | |
| Study design | 4 | Present key elements of study design early in the paper – this is done in the background on page 7 indicating that the study included quantitative data relating to fidelity and qualitative data relating to experience of study store managers (the COREQ checklist has been completed and submitted for the qualitative component) |
| Setting | 5 | Describe the setting, locations, and relevant dates, including periods of recruitment, exposure, follow-up, and data collection – these are covered in the methods section on pages 7 to 12 |
| Participants | 6 | (*a*) Give the eligibility criteria, and the sources and methods of selection of participants – the three quantitative components of the study are described on pages 8 and 9 and in figure 1 and table 1 |
| Variables | 7 | Clearly define all outcomes, exposures, predictors, potential confounders, and effect modifiers. Give diagnostic criteria, if applicable – variables are described in table 1 and on pages 8 to 10 |
| Data sources/ measurement | 8* | For each variable of interest, give sources of data and details of methods of assessment (measurement). Describe comparability of assessment methods if there is more than one group – covered in table 1 and on pages 8 to 10 |
| Bias | 9 | Describe any efforts to address potential sources of bias – survey development along with steps to minimise bias in data collection are covered on pages 9 and 10 |
| Study size | 10 | Explain how the study size was arrived at – this is covered in the methods on page 13. The participants in the survey were stores taking part in the WRAPPED study to which this observational process evaluation relates. |
| Quantitative variables | 11 | Explain how quantitative variables were handled in the analyses. If applicable, describe which groupings were chosen and why – this is covered in the data analysis section on pages 11 and 12 |
| Statistical methods | 12 | (*a*) Describe all statistical methods, including those used to control for confounding – see page 11 and 12 |
|  |  | (*b*) Describe any methods used to examine subgroups and interactions – not applicable in this study |
|  |  | (*c*) Explain how missing data were addressed – a small number of stores did not participate in the survey as outlined on page 13. Relevant analyses were based on the stores with complete data. |
|  |  | (*d*) If applicable, describe analytical methods taking account of sampling strategy – see pages 11 and 12 |
|  |  | (*e*) Describe any sensitivity analyses – not applicable in this study |
| Results | | |
| Participants | 13* | (a) Report numbers of individuals at each stage of study—eg numbers potentially eligible, examined for eligibility, confirmed eligible, included in the study, completing follow-up, and analysed – this is covered on page 13 |
|  |  | (b) Give reasons for non-participation at each stage – see page 13 |
|  |  | (c) Consider use of a flow diagram |
| Descriptive data | 14* | (a) Give characteristics of study participants (eg demographic, clinical, social) and information on exposures and potential confounders – this is not applicable here where the participants are study stores and managers completed a survey relating to the layout of the stores and location of products within them |
|  |  | (b) Indicate number of participants with missing data for each variable of interest – this is covered on page 13 where the small number of stores not participating in the survey is stated |
| Outcome data | 15* | Report numbers of outcome events or summary measures – numbers and percentages are reported for food items located in particular store areas |
| Main results | 16 | (*a*) Give unadjusted estimates and, if applicable, confounder-adjusted estimates and their precision (eg, 95% confidence interval). Make clear which confounders were adjusted for and why they were included – not relevant here for this study of store survey responses |
|  |  | (*b*) Report category boundaries when continuous variables were categorized – not applicable |
|  |  | (*c*) If relevant, consider translating estimates of relative risk into absolute risk for a meaningful time period – not applicable |
| Other analyses | 17 | Report other analyses done—eg analyses of subgroups and interactions, and sensitivity analyses – not relevant here |
| Discussion | | |
| Key results | 18 | Summarise key results with reference to study objectives – this is done at the start of the discussion on pages 20 and 21 |
| Limitations | 19 | Discuss limitations of the study, taking into account sources of potential bias or imprecision. Discuss both direction and magnitude of any potential bias – study limitations are covered on page 23 |
| Interpretation | 20 | Give a cautious overall interpretation of results considering objectives, limitations, multiplicity of analyses, results from similar studies, and other relevant evidence – this is covered in the discussion on 21 to 24 |
| Generalisability | 21 | Discuss the generalisability (external validity) of the study results – this is covered in the discussion on pages 24 and 25 |
| Other information | | |
| Funding | 22 | Give the source of funding and the role of the funders for the present study and, if applicable, for the original study on which the present article is based – the information is given on page 1 |

*Give information separately for exposed and unexposed groups.

**Note:** An Explanation and Elaboration article discusses each checklist item and gives methodological background and published examples of transparent reporting. The STROBE checklist is best used in conjunction with this article (freely available on the Web sites of PLoS Medicine at http://www.plosmedicine.org/, Annals of Internal Medicine at http://www.annals.org/, and Epidemiology at http://www.epidem.com/). Information on the STROBE Initiative is available at www.strobe-statement.org.
