# Supplementary material for: Implementation of a UK supermarket intervention to increase purchasing of fresh fruit and vegetables: process evaluation of the WRAPPED natural experiment
Source: Int J Behav Nutr Phys Act. 2024 Nov 11;21:128. doi: 10.1186/s12966-024-01679-3 (PMC11552182; doi:10.1186/s12966-024-01679-3)
Supplement: Supplementary file 3 — Supplementary Material 3 [file 12966_2024_1679_MOESM3_ESM.docx]

**Supplementary Table 1: Semi-structured interview guide – store staff**

| **Interview questions:**   1. **COVID-19**  - What impact has the coronavirus pandemic had on your Iceland store? - How have customers in your store reacted to the coronavirus pandemic? - What supply issues have you experienced as a result of the coronavirus pandemic?   - *How have fresh and frozen fruit and vegetable supplies been affected in particular* - How has the availability and positioning of food products changed as a result of the coronavirus pandemic?  1. **Experiences of Iceland staff involved in the study**  - How has the study impacted you and other staff?   - *How did the study change your workload? How did it impact your time and tasks?*   - *What worked well? What could have worked better?* - How do you think the study has impacted the company?   - *What benefits, if any, has the company gained?*   - *What do you think prompted the supermarket chain to take part in the study? (i.e. company’s social corporate responsibility agenda)*  1. **Product placement in Iceland supermarket**  - How do you think the layout of products in-store influences the products that customers buy?   - *What makes products near the front of the store, end-of-aisle and checkouts so tempting for customers?* - How do customers respond when healthy foods are placed near the front of the store, end-of-aisle and checkouts? How different is the response when the products are unhealthy or non-food? *(prompt for differences by location and product type)* - Who decides what products are placed in these locations? - How do you think the government’s intention of introducing restrictions on placing unhealthy products near the front of the store, end-of-aisle and checkouts will impact on sales in your store? And the food industry more generally? *(retailers and manufacturers)* - How much do you think where products are placed on the shelf influences customers’ choices?  1. **Perceptions about the role of supermarkets in supporting customers to buy more healthy products**  - What are the challenges for supermarkets in promoting healthy eating?   - *Prompts: supply chains, government legislation/policy, customer demands/needs, market research & data on trends* - What can supermarkets do to help customers to buy more healthy foods?   - *How does this vary between supermarkets?*   **End with:**   - What else would you like to share, or ask me about? |
| --- |

**Supplementary Table 2: Food items located in the checkout areas in the month preceding the intervention implementation survey completion**

|  | **Baseline** | | | | **1 month** | | | | **6 months** | | | |
| --- | --- | --- | --- | --- | --- | --- | --- | --- | --- | --- | --- | --- |
| **Outcome** | **Control** | **Inter-vention** | **P-value*** | **n** | **Control** | **Inter-vention** | **P-value*** | **n** | **Control** | **Inter-vention** | **P-value*** | **n** |
| Fruit | 3 (19%) | 1 (6%) | 0.34 | 33 | 1 (8%) | 0 (0%) | 1.00 | 24 | 1 (6%) | 1 (7%) | 1.00 | 35 |
| Snacking range (sugar coated fruit & nuts) | 15 (94%) | 17 (100%) | 0.49 | 33 | 11 (85%) | 10 (91%) | 1.00 | 24 | 18 (100%) | 17 (100%) | 1.00 | 35 |
| Sugar-free gum | 14 (88%) | 14 (82%) | 1.00 | 33 | 13 (100%) | 9 (82%) | 0.20 | 24 | 17 (94%) | 16 (94%) | 1.00 | 35 |
| Sugary gum/mints | 16 (100%) | 16 (94%) | 1.00 | 33 | 11 (85%) | 11 (100%) | 0.48 | 24 | 17 (94%) | 17 (100%) | 1.00 | 35 |
| Standard confectionery | 15 (94%) | 16 (94%) | 1.00 | 33 | 12 (92%) | 9 (90%) | 1.00 | 24 | 15 (83%) | 14 (88%) | 1.00 | 34 |
| Crisps | 1 (6%) | 0 (0%) | 0.49 | 33 | 0 (0%) | 0 (0%) | 1.00 | 24 | 3 (17%) | 2 (13%) | 1.00 | 34 |
| Hot drink products (tea/coffee) | 1 (6%) | 2 (12%) | 1.00 | 33 | 2 (15%) | 2 (18%) | 1.00 | 24 | 0 (0%) | 1 (6%) | 0.47 | 34 |
| Soft drinks | 1 (6%) | 1 (6%) | 1.00 | 33 | 2 (15%) | 3 (27%) | 0.63 | 24 | 4 (22%) | 4 (25%) | 1.00 | 34 |
| Water | 3 (19%) | 2 (12%) | 0.66 | 33 | 1 (8%) | 2 (18%) | 0.58 | 24 | 4 (22%) | 2 (13%) | 0.66 | 34 |
| Non-food items | 8 (50%) | 10 (59%) | 0.61 | 33 | 4 (31%) | 2 (18%) | 0.65 | 24 | 10 (56%) | 11 (69%) | 0.43 | 34 |
| Seasonal non-food items | 2 (13%) | 3 (18%) | 1.00 | 33 | 3 (23%) | 4 (36%) | 0.66 | 24 | 6 (33%) | 7 (44%) | 0.53 | 34 |

*P-values compare control and intervention at each time point. P-values are chi-squared except where data are too sparse (fewer than 80% of cells have an expected frequency greater than five, or not all cells have an expected frequency greater than one) in which case a two-sided Fisher’s exact test is used.

**Supplementary Table 3: Food items located in the aisle-ends opposite checkouts in the month preceding the intervention implementation survey completion**

|  | **Baseline** | | | | **1 month** | | | | **6 months** | | | |
| --- | --- | --- | --- | --- | --- | --- | --- | --- | --- | --- | --- | --- |
| **Outcome** | **Control** | **Inter-vention** | **P-value*** | **n** | **Control** | **Inter-vention** | **P-value*** | **n** | **Control** | **Inter-vention** | **P-value*** | **n** |
| Fruit | 0 (0%) | 1 (6%) | 1.00 | 27 | 0 (0%) | 0 (0%) | 1.00 | 22 | 0 (0%) | 0 (0%) | 1.00 | 31 |
| Snacking range (sugar coated fruit & nuts) | 0 (0%) | 4 (24%) | 0.13 | 28 | 3 (23%) | 3 (33%) | 0.66 | 22 | 2 (12%) | 3 (20%) | 0.65 | 32 |
| Sugar-free gum | 4 (36%) | 7 (41%) | 1.00 | 28 | 7 (58%) | 4 (44%) | 0.67 | 21 | 6 (35%) | 4 (27%) | 0.71 | 32 |
| Sugary gum/mints | 6 (55%) | 11 (65%) | 0.70 | 28 | 8 (62%) | 4 (44%) | 0.67 | 22 | 7 (44%) | 6 (43%) | 0.96 | 30 |
| Standard confectionery | 9 (75%) | 15 (88%) | 0.62 | 29 | 10 (77%) | 7 (78%) | 1.00 | 22 | 15 (83%) | 14 (88%) | 1.00 | 34 |
| Crisps | 5 (45%) | 1 (6%) | 0.02 | 28 | 2 (15%) | 0 (0%) | 0.49 | 22 | 3 (18%) | 2 (14%) | 1.00 | 31 |
| Hot drink products (tea/coffee) | 1 (9%) | 1 (6%) | 1.00 | 28 | 0 (0%) | 0 (0%) | 1.00 | 22 | 1 (6%) | 0 (0%) | 1.00 | 31 |
| Soft drinks | 5 (45%) | 3 (18%) | 0.20 | 28 | 0 (0%) | 0 (0%) | 1.00 | 22 | 4 (24%) | 4 (29%) | 1.00 | 31 |
| Water | 2 (18%) | 1 (6%) | 0.54 | 28 | 1 (8%) | 1 (11%) | 1.00 | 22 | 3 (18%) | 3 (21%) | 1.00 | 31 |
| Non-food items | 4 (36%) | 2 (12%) | 0.17 | 28 | 3 (23%) | 0 (0%) | 0.24 | 22 | 3 (18%) | 4 (29%) | 0.67 | 31 |
| Seasonal non-food items | 0 (0%) | 2 (13%) | 0.50 | 27 | 2 (15%) | 2 (22%) | 1.00 | 22 | 1 (6%) | 3 (21%) | 0.30 | 31 |

*P-values compare control and intervention at each time point. P-values are chi-squared except where data are too sparse (fewer than 80% of cells have an expected frequency greater than five, or not all cells have an expected frequency greater than one) in which case a two-sided Fisher’s exact test is used.

**Supplementary Table 4: Number (proportion) of stores offering health-related food promotions in the month preceding the intervention implementation survey completion**

|  | **Baseline** | | | | **1 month** | | | | **6 months** | | | |
| --- | --- | --- | --- | --- | --- | --- | --- | --- | --- | --- | --- | --- |
| **Stores offering health-related food promotions** | **Control** | **Inter-vention** | **P-value*** | **n** | **Control** | **Inter-vention** | **P-value*** | **n** | **Control** | **Inter-vention** | **P-value*** | **n** |
| Yes | 7 (47%) | 4 (27%) | 0.26 | 30 | 6 (50%) | 3 (30%) | 0.42 | 22 | 2 (14%) | 3 (21%) | 1.00 | 28 |

*P-values compare control and intervention at each time point. P-values are chi-squared except where data are too sparse (fewer than 80% of cells have an expected frequency greater than five, or not all cells have an expected frequency greater than one) in which case a two-sided Fisher’s exact test is used.
